# Testing a Novel Design Framework for Patient-Facing Machine Learning–Based Predictions of Heart Failure Decompensation

**DOI:** 10.1016/j.jacadv.2025.102171

**Published:** 2025-09-04

**Authors:** Meghan Reading Turchioe, So Hyeon Bang, Afra Shamnath, Stephanie Lai, Natalie Yee, Julie Lindmark, Jessica Federak, Viktoria Averina, David Slotwiner

**Affiliations:** a Columbia University School of Nursing, New York, New York, USA; b Boston Scientific Corporation, Marlborough, Massachusetts, USA; c Weill Cornell Medicine, New York, New York, USA

**Keywords:** artificial intelligence, cardiac implantable electronic devices, heart failure, machine learning, patient empowerment, patient engagement

## Abstract

**Background:**

Policies facilitating the return of personal health data mean there is an urgent need to investigate strategies to safely present machine learning–based predictions to patients.

**Objectives:**

The authors aimed to design and test the usability of a patient-facing smartphone application prototype displaying a predictive algorithm of cardiac decompensation to patients with cardiac implantable electronic devices (CIEDs).

**Methods:**

We created a design framework for presenting algorithm output and implemented it in high-fidelity prototypes of a smartphone app. Prototypes showed 3 conditions with varying degrees of change in cardiac decompensation risk: significant, moderate, or little to no change. We conducted a mixed-methods usability evaluation of the prototype at a large, urban health system. English-speaking adults with implanted, actively transmitting CIEDs participated in evaluation sessions. The primary endpoint was patient objective comprehension of the algorithm's output. Secondary endpoints were risk perception, behavioral intention, and information-seeking.

**Results:**

Twenty participants (mean age 54 years, 40% female, 65% White, and 35% Black or African American) completed the study. Comprehension of the algorithm output was high across conditions (80% to 85%), but comprehension of the algorithm's threshold varied (60% to 85%), as did comprehension of the CIED sensors contributing to the algorithm (63% to 93%). In response to the condition showing a significant change, the majority of participants would be “moderately” (40%) or “very” worried (30%) about worsening cardiac status, and 80% would call their doctor.

**Conclusions:**

The prototype demonstrated high levels of patient comprehension, appropriate risk perception, and behavioral intention, suggesting its viability for empowering patients with actionable health insights.

Machine learning (ML)–based predictions of clinical deterioration and other adverse events are increasingly common in clinical care.^1^ However, developing strategies for presenting patient-facing ML is becoming a more urgent issue. Cardiac implantable electronic devices (CIEDs) are one clinical context in which patient-facing ML may soon be implemented. CIEDs are medical devices that regulate cardiac rhythm and rate. Secondarily, they also remotely capture and transmit data on the heart's functioning and abnormal cardiac events to the care team, and can therefore serve as an early warning sign of decompensation and predict future adverse clinical events.^2^, ^3^, ^4^, ^5^

For the last decade, a growing consensus of patients with cardiac conditions requiring CIEDs, such as heart failure (HF), have been advocating for the return of their cardiac data.^6^ This mirrors the broader calls for the return of health data to patients and research participants, culminating in landmark policy changes such as the Information Blocking laws in the 21st Century Cures Act.^7^ In response, device manufacturers and clinicians alike have been openly discussing appropriate methods of returning data. Pertinent, unanswered questions include: What information do patients need to know, and what is superfluous? How should patient anxieties about cardiac status be balanced against their desire for information? What formats are optimal to ensure inclusion across patients' varying health literacy and numeracy levels? Should data summaries be aligned with clinician reports for ease of patient-clinician communication, or be tailored to patients' unique information needs, potentially at the expense of clinician explanations? For example, if clinicians see weekly summaries but patients want daily summaries, clinicians may struggle to support patients in interpreting their health reports. While specific to CIEDs, many of these questions extrapolate to broader questions being asked about returning other types of health data to patients.

Failure to answer these questions in a thoughtful manner can result in patient anxiety, patient and clinician frustration, and intervention-generated inequities.^6^^,^^8^ In the context of these unanswered questions, we partnered with a large CIED manufacturer, Boston Scientific Corp. Many Boston Scientific CIEDs feature the HeartLogic Heart Failure Diagnostic, an advanced tool for early detection of worsening HF.^9^ Details about the algorithm are provided in the Supplemental Appendix. Currently, the clinical teams caring for patients with Boston Scientific CIEDs (most often HF and cardiac electrophysiology specialists) receive weekly reports summarizing cardiac activity, a number of physiologic parameters measured continuously by the CIED (such as respiratory rate, heart rate, and heart sounds), and the HeartLogic Index.

We aimed to design and test the usability of a patient-facing smartphone application (app) prototype displaying HeartLogic Index and other CIED data to patients. The primary objective of this study was to assess patient objective comprehension of the ML-based HeartLogic Index. The secondary objectives were to assess risk perception, behavioral intention, and qualitative aspects of information-seeking within the prototype.

## Methods

### The “HeartLogic” application

#### Design

**Figure 1:**
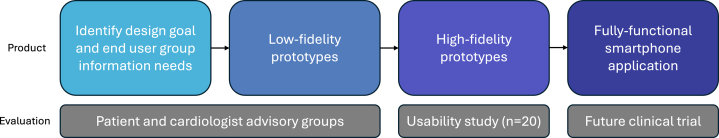
Design Process for Creating and Testing the HeartLogic App

**Figure 2:**
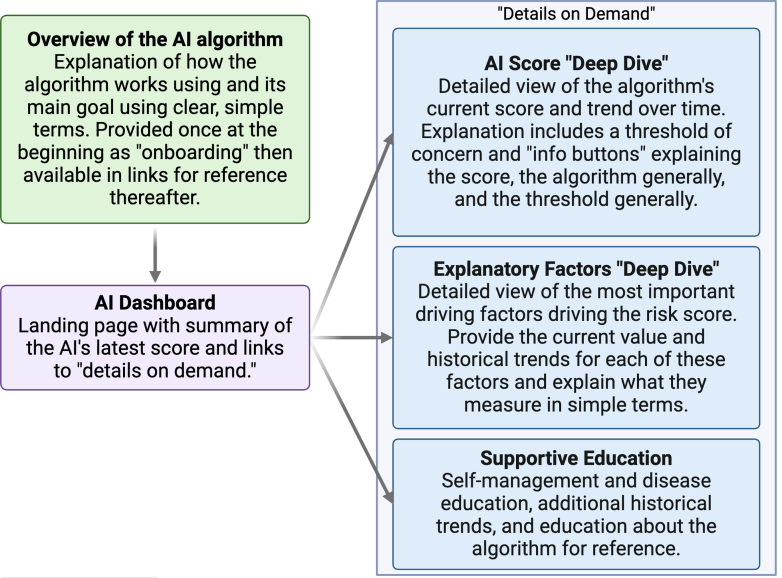
Design Framework for Communicating ML-Based Predictions to Patients AI = artificial intelligence; ML = machine learning.

The “HeartLogic” app's initial designs were developed through discussion among core research team members from Boston Scientific, Columbia University Irving Medical Center, and Weill Cornell Medicine/New York Presbyterian. The design process is described in detail in the Supplemental Appendix and summarized in Figure 1. We created a design framework, described in Figure 2, in which the algorithm is introduced in simple onboarding screens before bringing the user to the dashboard for the first time; this education is later made available through links that can be accessed from the dashboard. The dashboard contains an overview of the current HeartLogic Index number with links to view more details about the number, view score-driving features (ie, 5 CIED sensors), and view additional educational content.

#### Final prototypes

**Figure 3:**
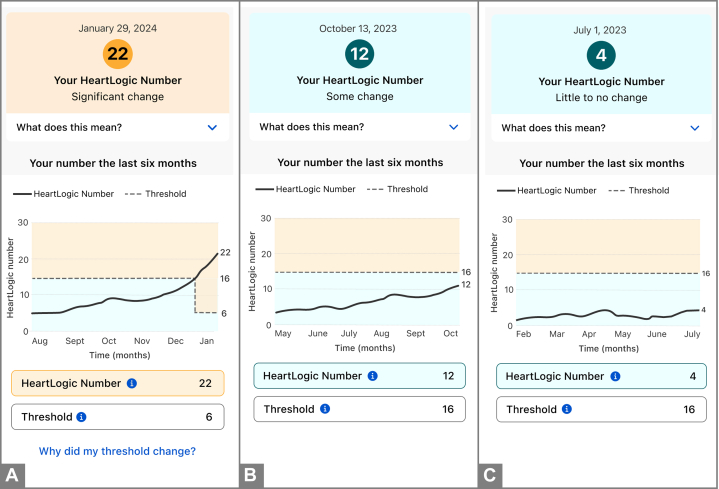
HeartLogic App Visualizations of the HeartLogic Index Showing a Significant Change, Some Change, and Little to No Change HeartLogic app visualizations of the HeartLogic Index with a significant change (A), some change (B), and little to no change (C).

The final app prototype included simulated patient data with the following subsections: 1) “onboarding” overview of the HeartLogic Index; 2) artificial intelligence dashboard; 3) HeartLogic Index “deep dive;” 4) CIED sensors (ie, explanatory factors) “deep dive; ” and 5) supportive education. We created 3 conditions of HeartLogic Index numbers, representing 3 possible HF states, for the evaluation: “significant change” with a number well above the threshold, “some change” with a number slightly below the threshold but trending upward, and “little to no change” with a low, stable number (Figure 3) so that we could measure the comprehension-specific task of classification.^10^ The line graph displays the trend of the HeartLogic index number over the prior 6 months, with the threshold. The “threshold” is an algorithmically derived number indicating the point at which clinicians should become concerned. Once a HeartLogic Index number crosses the threshold, the threshold drops lower as a conservative estimate of when the patient's number has dropped low enough again to indicate a true recovery.

### Sample and setting

We conducted a mixed-methods usability evaluation of the “HeartLogic” app, described below. Participants were recruited from a large, urban health system comprised of 11 hospitals in New York City. We partnered with the cardiac electrophysiology clinic, which is responsible for monitoring and managing patients with CIEDs, to obtain a list of all patients with Boston Scientific CIEDs across the health system. Eligibility criteria included: 1) implanted, actively transmitting Boston Scientific CIED with HeartLogic enabled; 2) age 18 years and older; 3) able to read and speak English; and 4) willing and able to participate in a remote usability session using Zoom. The Columbia University Irving Medical Center Institutional Review Board approved this study.

### Measures

The interview guide, integrating objective endpoints and semistructured interview questions, is provided in the Supplemental Appendix. The endpoints were anchored in Ancker et al's^11^ taxonomy for synthesizing the evidence on communicating numbers in health and mirror a prior study evaluating visualizations of ML-based predictions.^12^ This taxonomy offers a structured nomenclature for categorizing outcome measures evaluated across the studies, encompassing perceptions, decisions, and actions. It systematically organizes and describes these outcomes to enable consistent interpretation and comparison.

#### Primary endpoint

##### Comprehension of HeartLogic Index

The primary endpoint of this usability study was cognitive perceptions (ie, objective comprehension) of HeartLogic Index. Because the main task was classification, we measured this across the 3 HF conditions: significant change, some change, and little to no change. We adapted the questions from the International Organization for Standardization 9186 method, a recognized standard for assessing comprehensibility.^13^^,^^14^ We used the adapted comprehension question for each of the conditions, their respective thresholds, and each of the sensors.

##### Secondary endpoints

Affective perceptions (ie, risk perceptions–worry about worsening HeartLogic Index in a given condition and its impacts) were measured using a single validated item for each of the 3 conditions.^15^ Behavioral intention (eg, intent to act) was also measured for each of the 3 conditions using a validated item.^13^^,^^14^ Finally, we asked qualitative and survey questions about how participants navigated the prototype to seek additional information, in order to understand how they pursued explanatory information about HeartLogic Index and CIED sensors.

#### Sociodemographic and clinical characteristics

Participants completed a brief survey about their age, sex, race, ethnicity, education, and disability status. Additionally, they completed validated brief scales measuring digital literacy,^16^ health literacy,^17^ and subjective numeracy.^18^ The CIED device type was obtained from clinic records.

### Data collection

A research assistant (RA) contacted eligible patients by phone to describe the study and invite them to participate. Participants were purposively sampled to include a balance by age, gender, and race. Participants who agreed to participate were scheduled for a 60-minute virtual meeting with the RA and/or principal investigator (PI).

During the meeting, participants reviewed and signed the electronic consent form and completed baseline questionnaires using a local, secure instance of Qualtrics. The RA or PI then shared their screen showing the interactive prototypes. Participants were asked to take control of the screen to be able to independently interact with the prototypes. If they were unable to do so, they verbalized their intended interactions and the RA or PI completed clicking on their behalf.

A semistructured interview guide was used. Participants were first asked about their general experiences with HF, whether they had ever viewed their CIED data, and whether they had ever heard of “HeartLogic.” They were then advised to begin freely interacting with the prototype, verbalizing their train of thought following a cognitive walk-through methodology.^19^ Following the interview guide, participants were stopped on specific screens and asked a series of quantitative and qualitative questions. They viewed the “significant change” condition first, then “some change” and “little to no change” last; in sessions where time ran short, data on endpoints for the second and third conditions were recorded as missing. The entire session was audio recorded and transcribed verbatim.

### Data analysis

All quantitative and qualitative data originated from a single transcript of the usability session. The PI created a preliminary set of qualitative codes based on the key screens and functionalities of interest. Two RAs extracted participants' qualitative responses and answers to quantitative measures from the interview transcripts into a structured Qualtrics form. The PI reviewed all extracted information and confirmed its accuracy against the original transcript.

For the primary endpoint of comprehension, the 2 RAs and PI independently scored participant responses as correct or incorrect. The 3 independent scores were compared. Any discrepancies with less-than-perfect agreement were resolved through discussion. Then, this endpoint, all secondary endpoints, and demographic characteristics were summarized using basic descriptive statistics in R Studio.

Qualitative responses were synthesized from the data extraction form. The PI and 2 RAs identified subthemes by rigorously analyzing the codes that emerged from the data, following a general inductive approach^20^ to remain grounded in the participants' perspectives. To ensure methodological rigor, they maintained an audit trail to document decision-making processes and increase transparency.^21^ Additionally, they engaged in regular debriefing sessions to mitigate researcher bias and improve consistency across coders. Finally, perspectives were triangulated^22^ with quantitative findings to ensure that insights were well integrated the credibility of the identified subthemes was enhanced.

## Results

### Sample characteristics

Twenty participants completed the study. Their characteristics are presented in Table 1. Participants' mean age was 54.2 ± 18.3 years, 40% were female, 35% were Black or African American, and the majority were non-Hispanic/Latino (95%). Their education levels varied, with 35% having less than a Bachelor's degree. Most had moderate or high technology literacy as indicated by “agreeing” or “strongly agreeing” that they could use applications or computer programs (80%), video chats (75%), and troubleshoot technology issues (65%) on their own. Approximately half (55%) of the sample had inadequate health literacy.

**Table 1:** Sociodemographic Characteristics of the Sample (N = 20)

Age (y)	54.2 ± 18.3
Sex	
Female	8 (40%)
Male	12 (60%)
Race	
Black/African American	7 (35%)
White	13 (65%)
Ethnicity	
Non-Hispanic/Latino	19 (95%)
Prefer not to answer	1 (5%)
Device type	
Defibrillator	8 (40%)
Pacemaker	1 (5%)
Pacemaker/defibrillator	11 (55%)
Education	
Did not complete high school	2 (10%)
Some college or associate's degree	5 (25%)
Bachelor's degree	13 (65%)
Disability	
Yes	8 (40%)
No	12 (60%)
Ability to use apps/computer programs on own	
Strongly agree	9 (45%)
Agree	7 (35%)
Neutral	1 (5%)
Disagree	0 (0%)
Strongly disagree	3 (15%)
Ability to use video chat on own	
Strongly agree	7 (35%)
Agree	8 (40%)
Neutral	2 (10%)
Disagree	1 (5%)
Strongly disagree	2 (10%)
Ability to troubleshoot tech issues on own	
Strongly agree	8 (40%)
Agree	5 (25%)
Neutral	5 (25%)
Disagree	1 (5%)
Strongly disagree	1 (5%)
Health literacy	
Adequate	9 (45%)
Inadequate	11 (55%)
Subjective numeracy	14.0 ± 3.3

Values are mean ± SD or n (%).

Regarding the specific CIED type, 6 (30%) had cardiac resynchronization devices, and the remainder had Implantable Cardiac Defibrillators (ICD). Patients described their cardiac history that led to a CIED being implanted, which included major cardiac events, such as heart attacks, arrhythmias, and HF, that significantly impacted their health. They shared experiences of using various tracking methods, from devices like pacemakers and CardioMEMS (another heart failure monitoring system) to wearable technologies like Apple Watches, to monitor heart health. While some embraced lifestyle changes after their CIED was implanted, others maintained previous habits. The majority of patients had limited or no prior knowledge of HeartLogic, though a few were somewhat familiar with its functions, like monitoring fluid levels and heart rate.

### Primary endpoint: comprehension

**Figure 4:**
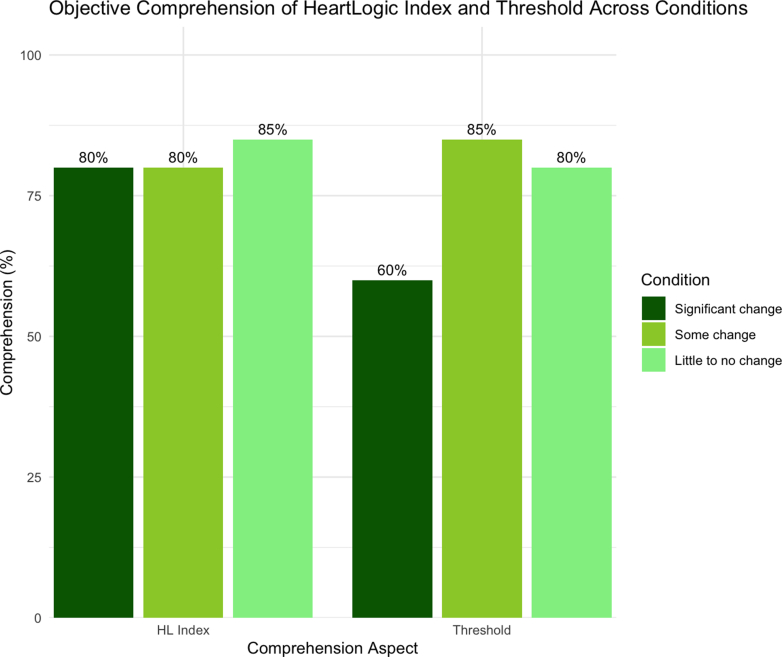
Comprehension of HeartLogic Index and Thresholds by Condition

**Figure 5:**
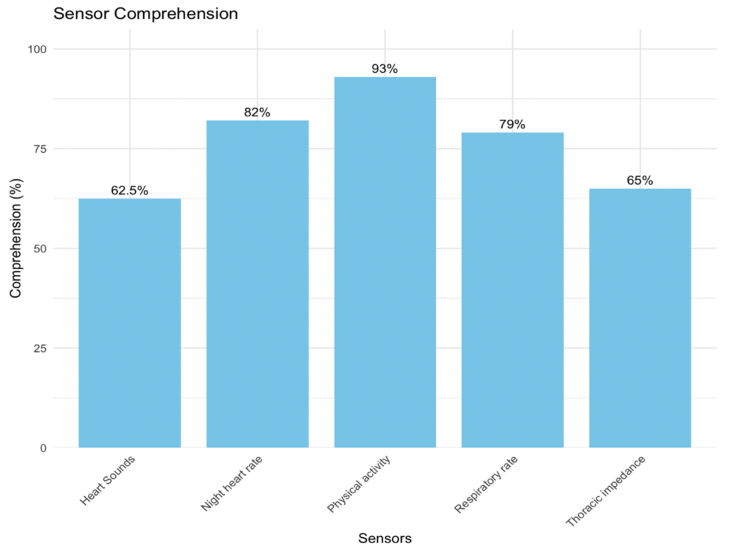
Comprehension of Individual CIED Sensors Missing responses by sensor: heart sounds, n = 4; night heart rate, n = 3; physical activity, n = 6; respiratory rate, n = 1; thoracic impedance, n = 3.

Objective comprehension results are shown in Figures 4 and 5, and associated illustrative quotes are shown for HeartLogic Index, the threshold, and for the individual sensors in Table 2. Comprehension of HeartLogic Index was high (80% to 85%) across all 3 conditions (significance, some, and little to no change). Comprehension of the threshold varied (60% to 85%) across the 3 conditions. Comprehension of the sensors ranged from 62.5% (heart sounds) to 93% (physical activity).

**Table 2:** Comprehension-Related Qualitative Themes' Summaries and Illustrative Quotes (N = 20)

Comprehension Task and Question	Qualitative Summary and Quotes	
	Comprehended	Did Not Comprehend
HeartLogic Index purpose (“How would you explain HeartLogic in your own words?”)	Participants understood that HeartLogic is a system that integrates data from multiple sensors to generate a numeric index, providing real-time insights into heart health, tracking changes in heart status, and assisting patients and health care teams in monitoring and managing heart failure progression. “*You have sensors that will keep monitoring your heart to see if your condition is worsening. And the information goes to your health care team. And they're going to decide what the next step may be. So, the benefit is it tracks your heart. It tells your heart team information that will help them in treating your condition. That's what I understood.*”*— Patient 14*	Participants expressed confusion about HeartLogic's functionality and personal relevance. “*It's like it keeps data to see how your heart rate is doing. And so it basically just keeps track of data, to see what's going on with you. It doesn't explain what that information tells you, but it tells you what it monitors. I would like to know more information on what HeartLogic means.*”*—Patient 1*
Significant change		
HeartLogic Index number (“What does the 22 mean on this screen?”)	HeartLogic numbers were perceived as indicators of heart health status, with higher values suggesting worsening conditions or deviations from normal thresholds, prompting patients to monitor their status closely and consult their health care providers when thresholds are exceeded. “*This is my current HeartLogic number that is based on those 5 variables that my device has sent.*”*—Patient 7*	Participants understood that HeartLogic numbers are metrics tracking changes in heart health over time, but expressed confusion about the significance of specific values, the parameters defining thresholds, and how these relate to their individual conditions. “*It looks like it just means like the average number for the month.*”*— Patient 3*
HeartLogic Index former threshold (“What does the 6 mean on this screen?”)	About half of the participants understood that HeartLogic thresholds are used to indicate significant changes in heart health, but even those who correctly comprehended the former threshold expressed confusion about why thresholds shift and how these changes impact their understanding of heart failure management. “*I don't know why it goes down like that. Why would the threshold go lower than if the threshold was 15 or 16, why would it go to 6? What does that mean?*”*—Patient 8*	Many participants struggled to interpret HeartLogic graphs and the significance of values like 16 and 22, with confusion surrounding the relationship between numbers, thresholds, and their implications for heart health management. “*I have no idea. It's some level measuring up to the 22; to the midpoint between 6 and 22, 16 and 6 is 22, so it's midpoint.*”*—Patient 17*
HeartLogic Index new threshold (“What does the 16 mean on this screen?”)	Similarly to the former threshold, those who correctly comprehended the new threshold questioned the lowering of HeartLogic thresholds, from values like 16 to 6, expressing confusion about the rationale behind these changes and how the thresholds relate to their activity levels and heart health status. “*It's the threshold number that is being measured, and where they want me to be, I guess? I don't know what happened here that it dropped, you know, from maybe 15 to 5, but I don't know why that happened.*”*—Patient 13*	Many participants expressed confusion about baseline values, threshold changes, and the rationale behind lowering thresholds as HeartLogic numbers increase, often highlighting stress and uncertainty in interpreting these shifts and their implications for heart health management. “*Why does it become 6? It looks like maybe at the beginning was a 6. Suddenly, it was okay to be a 15, and now it's actually not. That's a little confusing to me. How long are you across that threshold before you're in contact with your doctors? That could be very stressful.*”*—Patient 7*
Some change		
HeartLogic Index number (“What does the 12 mean on this screen?”)	Most patients understood that 12 was the current HeartLogic number and that it is below the threshold of 16, associating it with stability but expressing concerns about its upward trend, uncertainty about the implications of movement toward the threshold, and a need for further clarity on its significance for heart health. “*The HeartLogic number is at 12, which is lower than the threshold, so it's still with some changes.*”*—Patient 20*	The few participants who did not comprehend viewed the HeartLogic number 12 with varying levels of concern, often struggling to interpret its significance, noting that it is higher than normal for some but lower than extreme values like 22, while others find the graph insufficiently clear to provide meaningful context. “*That [12] would be just another average number for another month or so. It's like the graph is not defined enough.*”*—Patient 3*
HeartLogic Index threshold (“What does the 16 mean on this screen?”)	Participants understood that 16 was the threshold, with many associating crossing it with the need for medical action, though some questions remain about why this threshold did not change. “*The 16 is the danger zone.*”*—Patient 15*	Patients expressed a lack of clarity in interpreting HeartLogic numbers, often describing them as insufficiently explained, arbitrary, or devoid of meaningful context to guide understanding or action. “*That's the national average, I guess.*”*—Patient 12*
Little/no change		
Heart Logic Index number (“What does the 4 mean on this screen?”)	Participants viewed the HeartLogic number 4 as a reassuring indicator of stability, though some express that the number itself lacks meaningful context unless paired with symptoms or additional explanations. “*It's telling me that my HeartLogic is basically the same. Again, the number 4 is what my HeartLogic is. Virtually nothing changed since February.*” *—Patient 2*	Participants expressed a desire for more detailed explanations of HeartLogic numbers, with some associating changes with minimal impact, while others highlight the need for clarity about which specific health parameters contribute to their status. “*I'm very low. So, I don't want to go any lower. But again, I don't know what's wrong with me, but it could be a combination of a few things. Well, I would love to see an explanation, which [sensor] is out of whack.*”*—Patient 12*
HeartLogic Index threshold (“What does the 16 mean on this screen?”)	Participants recognized 16 as a critical HeartLogic threshold indicating a point of concern or potential intervention, with values below it, such as 4, generally viewed as stable or controlled. Some expressed uncertainty about the implications of changes and the directional meaning of the values relative to heart health. “*If 16 is the norm or is the warning area in any one of those and I am only at 4 from 2, I don’t think that would make any dramatic action…make for any dramatic action.*”*—Patient 8*	Some participants mistakenly perceived the threshold of 16 as a comparative or average benchmark but express a need for more contextual information to understand its significance. “*Little or no change. That sounds good. The number doesn't mean anything to me, although compared to 22, it's significantly smaller.*”*—Patient 17*
Sensors		
Respiratory rate (“What is being measured on this page?”)	Participants understood respiratory rate as the number of breaths per minute tracked over time, with some associating it with heart or lung health and recognizing its variability with activity levels. “*My respiratory rate how many breaths per minute I take.*”*—Patient 17*	Some participants expressed confusion and lack of context in interpreting respiratory rate data, recognizing its potential connection to heart failure but emphasizing the need for guidance from a cardiologist to make the information meaningful and actionable. “*There's no context, but it was the same, went down a little bit and then it went way up. What breathes per minute. I have no idea why, but if that chart actually said it went from 22 average to 35 or something like that was up 60/70 percent, I would tell the doctor. I would have no idea what it means, so I would immediately tell the doctor.*”*—Patient 8*
Night heart rate (“What is being measured on this page?”)	Participants understood night heart rate as a measure of resting heart rate during sleep, recognizing its potential to reveal irregularities or changes that may indicate health concerns. “*The night heart rate is probably while you're asleep, and they would probably want to know that because it's more of a calm time. It's more of a baseline rate. You know, you're not running upstairs or sitting in the car, or anything like that, and is probably the baseline that it's looking at.*”*— Patient 13*	Some participants expressed misunderstandings about night heart rate data or failed to understand how it differed from a general heart rate measure. “*This is how many pulses you're having per minute.*”*—Patient 9*
Physical activity (“What is being measured on this page?”)	Participants perceived activity-level monitoring as a measure of physical engagement, associating it with daily routines, exercise, or inactivity, and acknowledge its potential to signal worsening heart failure. However, many express uncertainty about how it distinguishes between types of activity. “*It's just measuring how much you're up and doing stuff throughout the day. If it notices a trend that you're not doing as much, it will graph that accordingly, and then start to lower your number which will raise a red flag for somebody either me or my doctor.*”*—Patient 18*	A small number of participants conflated physical activity monitoring with heart functioning. “*Is it the activity of your heart? Your heart is always active.*”*—Patient 2*
Thoracic impedance (“What is being measured on this page?”)	About half of participants understood thoracic impedance as a measure of how easily electricity flows through the chest, often linking it to fluid buildup in the lungs or changes in heart function, though some express confusion about its technical details. “*It basically just tells you how easy electricity flows through your chest and what they call the electricity to add more resistance and less resistance. So, if you have more fluid in your lungs, it can cause less electricity to flow. And, I'm guessing it has to do with the shock you might get from one of these devices.*”*—Patient 4*	Many participants express confusion about this concept, or conflated it with heart rhythm. “*I believe that's dealing with the rhythm of your heart, if I'm not mistaken.*”*—Patient 1*
Heart Sounds (“What is being measured on this page?”)	About half of the participants comprehended heart sounds, often linking them to rhythm or changes related to heart failure. “*I correlate it with the ultrasound sound. Like when I go for my echo.*”*—Patient 11*	Many participants expressed uncertainty about specific terms like “S3,” struggled to interpret auditory descriptions like “whoosh,” and question the relevance of this information to the average individual without clear context. “*I don't know. It says S3. What is S3? I mean, I don't know, like I said, you never hear your heart. I don't know what a whoosh noise is, except whoosh is like the wind, you know, air bursting through. That's what I think whoosh is.*”*—Patient 12*

### Secondary endpoints: risk perception, behavioral intention, and information-seeking

Risk perception and behavioral intention results and associated illustrative quotes are shown in Table 3. In response to the condition showing a significant change, the majority of participants would be “moderately” (40%) or “very” worried (30%) about worsening HF, and 80% would call their doctor. In response to the condition showing some change, the majority would be “slightly” (35%) or “not at all” worried (30%), 45% would call their doctor, 20% would do nothing, and 20% would take a different action such as attempt to self-manage. In response to the condition showing little to no change, most were “not at all” worried, and half would do nothing, though a small number of people would call their doctor or go to the hospital (15%).

**Table 3:** Risk Perception and Behavioral Intention Quantitative Results and Qualitative Themes' Summaries and Illustrative Quotes From the Sample (n = 20)

	Significant Change	Some Change	Little/No Change
Risk perception			
Quantitative evaluation: “Based on this information, how worried are you about worsening heart failure?”			
Very worried	6 (30%)	0	1 (5%)
Moderately worried	8 (40%)	3 (15%)	1 (5%)
Slightly worried	1 (5%)	7 (35%)	1 (5%)
Not at all worried	4 (20%)	6 (30%)	12 (60%)
Missing	0	4 (20%)	5 (25%)
Qualitative summary and quotes	Participants expressed varying levels of concern regarding changes in heart-related metrics, influenced by their understanding of the data, presence of symptoms, and access to medical guidance. Many emphasized the importance of clinician interpretation, reliable devices, and actionable thresholds to manage their responses effectively. “*I am someone who, like all heart patients…it's your heart; there is a lot of symbolic charge to that organ to know that something bad is happening, which is what that number tells you, and not have contact with a reassuring medical figure, I think that would be worrisome.*”*—Patient 7*		
Behavioral intention			
Quantitative evaluation: “What would you do if you saw this information?”			
Go to the hospital/emergency room	1 (5%)	0	1 (5%)
Call my doctor	16 (80%)	9 (45%)	2 (10%)
Do something else (eg, self-manage)	1 (5%)	4 (20%)	1 (5%)
Do nothing	1 (5%)	4 (20%)	10 (50%)
Missing	1 (5%)	3 (15%)	6 (30%)
Qualitative summary and quotes	Participants expressed a strong preference for seeking immediate clarification from health care professionals when observing concerning trends or unfamiliar readings in their health data. While most indicated they would contact their doctor or specialist promptly, their level of concern and actions varied based on symptoms, perceived severity, and the need for professional interpretation of the data. “*No, I would call my doctor first. If I feel fine, I'm breathing fine, I don't feel any irregular heartbeats or anything like that, I would call my doctor. But if it feels like you know I'm not sleeping well or like I'm having a hard time breathing or anything like that. If I have these advanced heart failure symptoms, then I would go to the ER.*”*—Patient 14*		

Survey responses and associated illustrative quotes relating to information-seeking within the app are shown in Table 4. Few participants clicked (without being prompted by the researcher) on explanations about how to interpret the HeartLogic Index (35%), the changing threshold (30%), and the individual sensors (25%), or the information buttons defining the index (20%) and threshold (20%).

**Table 4:** Risk Perception and Behavioral Intention Quantitative Results and Qualitative Themes' Summaries and Illustrative Quotes From the Sample (N = 20)

Measurement	Question	Results
Quantitative measurement of navigation	Initial click after dashboard	Details about the HeartLogic number: 8 (40%) Score-driving features (ie, CIED sensors): 3 (15%) Additional educational content: 6 (30%) Missing or N/A^a^: 3 (15%)
	Did they click on “What does this mean” info button without prompting?	Yes: 7 (35%) No: 12 (60%) Missing or N/A^a^: 1 (5%)
	Info button: Did they click on the HeartLogic Number info button without prompting?	Yes: 4 (20%) No: 15 (75%) Missing or N/A^a^: 1 (5%)
	Did they click on the threshold info button without prompting?	Yes: 4 (20%) No: 15 (75%) Missing or N/A^a^: 1 (5%)
	Did they click on the “Why did my threshold change” info button without prompting?	Yes: 6 (30%) No: 13 (65%) Missing or N/A^a^: 1 (5%)
	Did they click on the “What is being measured?” info button without prompting?^b^	Yes: 5 (25%) No: 8 (40%) Missing or N/A^a^: 7 (35%)
Qualitative themes summaries and illustrative quotes	HeartLogic video	Participants expressed a range of attitudes toward using videos for learning about HeartLogic: while some patients expressed interest, particularly if the video offers practical examples or specific guidance, others preferred reading or self-navigation, citing disinterest or skepticism about the value of videos. “*I know it's a device that can monitor my heart. I know that's for a fact. In the video, I want to see exactly how it works.*”*—Patient 19*
	HeartLogic info buttons	Participants expressed varied views about reading and revisiting information, with some valuing detailed written content for learning or initial reference, while others preferred graphs or personalized metrics over text, citing the redundancy of repeated information and the tendency not to revisit static resources frequently. Overall, even the participants who would not refer to the info buttons still felt the information should be there. “*I probably wouldn't go back to it, but I think it's something that needs to always be included.*”*—Patient 9*
	Historical trends	Participants expressed varied preferences for reviewing data, with some valuing detailed formats like spreadsheets or trend analysis for identifying patterns, while others preferred simpler lists for ease of understanding and tracking changes. Some participants highlighted the importance of contextualizing data with specific dates, times, and activities to understand anomalies, while others mentioned challenges with overly complex or insufficiently clear data presentations. “*I would like to be able to really drill into the data, to look into those dates, and trends over those dates…It's more when I'm looking at the numbers, to be able to compare them, how they're changing.*”*—Patient 2*
	Colors	Participants expressed mixed opinions on the use of colors in data presentation, with some favoring clear, intuitive indicators like red for alerts and blue for stability, while others found the current palette bland or unclear and suggested brighter, more distinct colors and better labeling to enhance clarity and usability. “*I don't particularly like the colors. I can see why red was avoided–red is the color of your heart and the color of alarm, so perhaps not red. The pale green and pale yellow or beige seems a little anemic to me. I would go with something brighter.*”*—Patient 7*
	Self-management: diet and exercise	Participants expressed a desire for personalized recommendations in diet, weight, and exercise management, emphasizing that such guidance should cater to individual needs rather than generic advice. While some valued trusted resources for heart-healthy recipes or general tips, others sought specific, actionable plans or access to a health care professional, such as a nutritionist, to create tailored approaches based on their unique health conditions and lifestyles. “*I would spend time on this. Diet and weight help with your condition. Exercise, my doctor advised me to exercise.*”*—Patient 14*
	Self-management: emotions and mental health	Participants expressed mixed views on addressing emotional support, with some emphasizing its importance, particularly in adapting to life changes or managing family-related stress, while others felt it was less relevant to their needs or preferred to rely on personal coping strategies. A few participants highlighted the value of learning about emotional well-being as part of a holistic approach to managing heart failure, though some preferred such discussions to occur with their primary doctor rather than within a broader heart failure program. “*You should have a therapist. Because at first, your life changes drastically. You can't do what you used to do. You're limited.*”*—Patient 1*
	Design gaps and suggestions	Participants expressed varied preferences for app usage, with some intending to check it weekly or less frequently over time, while others emphasized the importance of features like viewing heart rhythms, providing proxy access for family members, and receiving timely notifications about significant changes. They also highlighted the need for clearer explanations of metrics and thresholds to better interpret the data and its implications for their health. “*I would probably look at it every couple of weeks, and just see where I'm at. I'm not one to sit there and stare at this stuff every day. I just live my life, and what happens, happens. And so I'm not going to become obsessed with it or anything. The first couple of weeks, I would see how it's going. And then after that, probably every few weeks. You should always still be alerted by the app, since you have it. I think that would be a good. An app push notification or just an email, just saying you're having an increase in your HeartLogic number this month or this week.*”*— Patient 9*

a Marked N/A in the case where the researcher took over navigation of the prototype due to time constraints.

b Recorded for the specific sensor detailed view they opened first.

## Discussion

In the study, we aimed to conduct development and usability testing of a patient-facing smartphone app displaying CIED data and specifically a proprietary algorithm, HeartLogic, with the primary endpoint being objective comprehension (eg, correct classification) of the HeartLogic Index. In designing the app prototype, our team weighed patients' desire for detailed information about their heart status and the imperative for information to be presented clearly and concisely against the possibility that a given design choice may deliver an inappropriate amount of information, or in the wrong format, and ultimately cause patient anxiety and confusion. The app framework presenting information in a “details on demand” format (Figure 1) paired with colored line graphs depicting the HeartLogic Index's trends over time led to overall high comprehension and appropriate risk perception and behavioral intention in this small but diverse sample, suggesting it may be a useful approach for future work (Central Illustration).

**Central Illustration:**
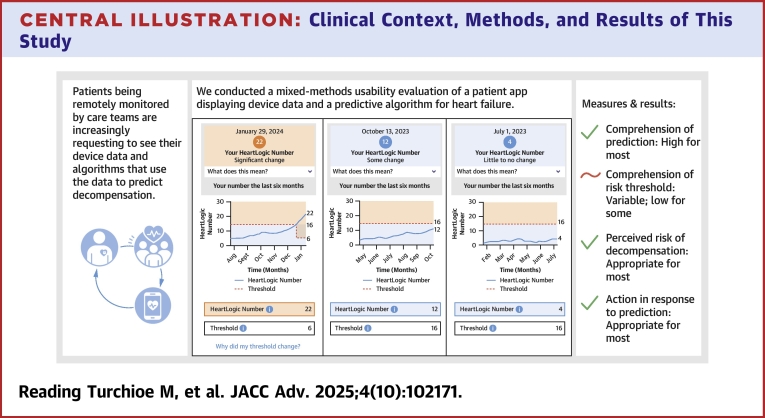
Clinical Context, Methods, and Results of This Study CIED = cardiac implantable electronic device.

However, we also noted several areas for further refinement and continued research. Specifically, many patients were confused about changing risk thresholds—a necessary patient safety feature designed by data scientists with cardiologists as the end user in mind. This exemplifies the challenges of extending ML-based predictive models user groups to patients when they were not the original intended audience. In other adjacent areas, we have already seen how information communication needs clash with model optimization choices; numerous studies report that clinicians and patients prefer explainable ML that is slightly less accurate over more accurate but nonexplainable ML.^23^^,^^24^^,^^25^, ^26^, ^27^

Another challenge was balancing patients' desire to understand the next steps after a “significant change” in HeartLogic Index against the strong preferences of health care professionals to manage patients above the threshold with their own practice-established workflows, thus making universal guidance nearly impossible. In fact, many patients reported their “next step” would be to call their doctor across all 3 conditions, reinforcing well-documented health care professionals' concerns about the exchange of patient-generated data with patients leading to an inundation of patient phone calls.^28^ In the theoretical use case, all practices would still receive alerts and detailed reports about patients above the threshold, so patients would not need to contact their care teams to inform them about the alert, though some may feel more comfortable doing so regardless. Moreover, health care professionals could educate patients during their first phone call, which patients agreed would mitigate their concerns and reduce their future outreach to their care team. Future work can explore language recommending HF self-care behaviors that should universally be practiced, but this general guidance may still fall short of the clear, explicit directions patients want. Ultimately, explicit conversations between patients and health care professionals prior to accessing an app containing ML-based predictions may be one of the most effective tools to establish appropriate lines of communication in the event of an abnormal HeartLogic Index number.

This study is among the first to explore strategies for returning ML-based predictions directly to patients through a mobile app. Most studies on remote monitoring (RM)-based CIED systems have focused on understanding patients' perceptions, attitudes, and communication expectations. Patients generally express strong interest in CIEDs and accompanying smartphone apps designed to assist with health management. For instance, over 60% of HF patients reported interest in apps that offer features such as activity tracking, symptom management, and medication reminders.^29^ Apps like HeartMapp received positive feedback, with patients expressing confidence in their ease of use and real-time data capabilities.^30^ However, concerns about data security, battery life, and the reliability of CIED data persist, with some patients expressing frustration when RM data did not align with their actual health outcomes.^31^ While many patients appreciate the convenience and independence of CIED data being remotely transferred to their health care team, others still prefer in-clinic visits for personalized care and reassurance from face-to-face interactions with health care providers.^32^

Recent studies have emphasized that how RM data are presented play a crucial role in enhancing patient understanding and engagement. Patients have expressed a strong preference for user-friendly data visualizations, such as color-coded bar charts and icons, which make complex medical information more accessible and easier to interpret.^33^ For example, HF patients and their caregivers reported that real-time status updates, particularly those using visual cues like color-coded “green” indicators for stable conditions, provided reassurance and a better sense of control over their health.^34^ Furthermore, participants in dashboard design sessions have highlighted the need for intuitive visual aids, such as flashing indicators to signal when to contact a physician, and simple symbols to represent device functions.^35^

### Strengths and limitations

Strengths of this study included the human-centered design approach that anchored the prototype design, the recruitment of a diverse sample of patients with HeartLogic-enabled devices with respect to age, sex, race, and health literacy, and the use of validated measures of comprehension and other endpoints anchored in a previously published taxonomy.^11^ Limitations of this study included the small size and high technology literacy of the sample, which may have skewed results toward higher usability. While our findings suggest high levels of comprehension, the small sample size results in wide CIs around these estimates (eg, 80% comprehension corresponds to a 95% CI of approximately 62% to 98%), limiting the certainty with which we can generalize these results. Additionally, our sample was slightly younger but more diverse in terms of race and sex when compared to larger device trials for Boston Scientific CIEDs (MULTISENSE clinical trial^9^ and postmarket validation studies^36^^,^^37^) and CardioMEMS trials (GUIDE-HF clinical trial,^38^ MONITOR-HF clinical trial^39^). This may have contributed to the high perceived usability of the app; older adults have unique needs with respect to usability and usefulness of health technologies.^40^ Thus, larger clinical validation studies maintaining the diversity of our sample while testing the app in older adults are needed in the future.

## Conclusions

In this study, we highlight the potential for patient-facing applications to effectively communicate ML-based predictions from CIEDs, such as the HeartLogic Index, while balancing the complexities of usability and comprehension. The app prototype demonstrated high levels of patient comprehension, appropriate risk perception, and behavioral intention, suggesting its viability for empowering patients with actionable health insights. However, challenges remain in addressing misunderstandings about dynamically changing thresholds and aligning patient preferences with clinician workflows. Future work should focus on refining communication strategies, tailoring guidance for diverse patient needs, and fostering preemptive clinician-patient dialogues to optimize the integration of patient-facing ML tools into routine care. This study underscores the importance of inclusive, user-centered design in advancing equitable and effective health technologies.

Perspectives **COMPETENCY IN PATIENT CARE AND PROCEDURAL SKILLS:** This study demonstrates that patient-facing ML predictions for HF decompensation can be effectively communicated using structured visualizations and a “details on demand” approach, enhancing patient comprehension and engagement. However, challenges remain in explaining dynamic risk thresholds, necessitating further research on optimizing educational strategies and aligning patient-accessible ML outputs with clinician workflows.

**TRANSLATIONAL OUTLOOK:** Future studies should evaluate the real-world impact of ML-based applications on patient behavior, health care utilization, and clinical decision-making, ensuring seamless integration into cardiovascular care.

## Funding support and author disclosures

This project was funded by a research grant from 10.13039/100008497Boston Scientific. Dr Turchioe also reports funding from the 10.13039/100000056National Institute of Nursing Research (NINR; R00NR019124). Drs Yee, Lindmark, Federak, and Averina are employed by Boston Scientific. Dr Turchioe has received consulting fees from Boston Scientific; is a co-founder of Iris OB Health Inc; and holds equity in Iris OB Health Inc. All other authors have reported that they have no relationships relevant to the contents of this paper to disclose.
