# Supplementary material for: Testing a Novel Design Framework for Patient-Facing Machine Learning–Based Predictions of Heart Failure Decompensation
Source: JACC Adv. 2025 Sep 4;4(10):102171. doi: 10.1016/j.jacadv.2025.102171 (PMC12791880; doi:10.1016/j.jacadv.2025.102171)
Supplement: Supplemental File [file mmc1.pdf]

“Testing a novel design framework for patient-facing machine learning-based predictions of heart failure decompensation”

Supplementary Methods

A. HeartLogic Index: Machine learning algorithm development

Developer: Boston Scientific Corp. (Marlborough, MA).

Description: Many Boston Scientific CIEDs feature the HeartLogic™ Heart Failure Diagnostic, an advanced tool for early detection of worsening heart failure. Developed using ML and traditional algorithmic techniques, HeartLogic is comprised of a composite trend called the HeartLogic Index and a configurable yellow alert. The HeartLogic Index is computed daily by analyzing device-based measurements of heart sounds, thoracic impedance, respiration, night heart rate and activity. It measures changes from a historical baseline in a worsening direction and weights those changes based on a patient’s current risk level. Baselines are assessed based on data spanning up to three recent months. An alert is issued when the HeartLogic index crosses above a set threshold.

Validation: In the prospective validation of the HeartLogic algorithm on 400 CRT-D patients, it was shown to have 70% sensitivity at 1.56 alerts-per-patient-year false positive rate with a median warning of 34 days prior to a heart failure event.[1] Several subsequent post-market studies in CRT-D and ICD patients in various geographies found a similarly high performance with high observed sensitivity of 69-100% and a median of 34-38 days.[2,3]

Current state: Currently, the clinical teams caring for patients with Boston Scientific CIEDs (most often heart failure and cardiac electrophysiology specialists) receive weekly reports summarizing cardiac activity, a number of physiologic parameters measured continuously by the CIED (such as respiratory rate, heart rate, and heart sounds), and the HeartLogic Index.

B. Design process

Design goal: The design goal was to communicate to users when the HeartLogic Index was in a normal versus abnormal range relative to the established threshold number. Secondarily, we also sought to answer users' questions about *why* a HeartLogic Index was or was not elevated by providing additional contextual details about the algorithm and what was driving its value.

Iterative feedback: Through several meetings spanning over a year, a professional designer sketched lower- and then higher-fidelity prototypes of individual screens, options for visualizing HeartLogic Index, and eventually an app flow between screens. Boston Scientific convened patient and cardiologist advisory groups throughout the year to solicit feedback on the evolving prototypes. Feedback was summarized and shared with the core research team for further discussion and refinement of the prototype.

Evidence: For the central visualization displaying HeartLogic Index, we consulted the evidence based on studies evaluating information communication formats more broadly, with the goals of beginning with designs that were evidence-based with respect to high objective comprehension across diverse older adults with varying literacy and numeracy levels.[4–6] In particular, colored number lines have shown success in communicating abnormal ranges for laboratory values in prior work[7] and recent work shows they may be effective in communicating ML-based predictions.[8] However, early feedback from the patient and cardiologist advisory group indicated that visualizing longitudinal data on the score would be more useful in interpretation than single time points. One important reason for this is that the cardiologist's report contains longitudinal data, and the advisory groups felt that concordance between the cardiologist and patient views should be prioritized. Given the dearth of studies examining how to show ML-based predictions' trends over time, we opted to test a simple line graph that used colors and labels to enhance interpretation.

Design framework: For the overarching app prototype, we embraced the "details on demand" techniques that have been employed for decades in the data visualization field to selectively provide information to users in sequential, on-demand formats rather than all at once to avoid information overload.[9,10] This was intended to accommodate the wide range of preferences that patients have expressed about the level of detail that should be provided alongside a risk score.[11–13] We created a framework for providing these details as described in Figure 1, in which the algorithm is introduced in simple onboarding screens before bringing the user to the dashboard for the first time; this education is later made available through links that can be accessed from the dashboard. The dashboard contains an overview of the current HeartLogic Index number with links to view more details about the number, view score-driving features (i.e., five CIED sensors), and view additional educational content.

Final prototypes: High-fidelity, interactive prototypes were created using Figma prototyping software. The final app prototype included *simulated* patient data with the following sub-sections: (1) "onboarding" overview of the HeartLogic Index, (2) AI dashboard, (3) HeartLogic Index "deep dive," (4) CIED sensors (i.e., explanatory factors) "deep dive," and (5) supportive education.

- First, users are guided through a series of educational screens explaining what HeartLogic Index is in plain language and what the purpose of the app is. One of the screens included a representation of a brief, educational video explaining HeartLogic Index.
- The user then views a dashboard displaying (1) a summary of the current HeartLogic Index number, linking to a detailed view page, (2) a button to link to individual sensor data detailed views, and (3) additional resources containing general information about the HeartLogic Index algorithm, self-management education (i.e., managing diet, physical activity, and mental health), and a historical list of previous HeartLogic Index summaries for each week. The app data was intended to be updated weekly.
- The HeartLogic Index "deep dive" page shows a summary of the HeartLogic Index number, a line graph, and additional links to information modals.
- We created *three conditions* of HeartLogic Index numbers, representing three possible heart failure states, for the evaluation: "significant change" with a number well above the threshold, "some change" with a number slightly below the threshold but trending upward, and "little to no change" with

a low, stable number so that we could measure the comprehension-specific task of classification.[14] The line graph displays the trend of the HeartLogic index number over the prior six months with the threshold. The “threshold” is an algorithmically derived number indicating the point at which clinicians should become concerned. Once a HeartLogic Index number crosses the threshold, the threshold drops lower as a conservative estimate of when the patient’s number has dropped low enough again to indicate a true recovery.

- The CIED sensors “deep dive” shows a list of five sensors that drive HeartLogic Index: respiratory rate, night heart rate, activity level, thoracic impedance, and heart sounds. The initial view shows a weekly average of each sensor, and contains links to click to view each sensor in greater detail with a written, plain language explanation of what the sensor measures and a line graph trend of the sensor’s measurements over the prior six months.

C. Moderator's Guide

<u>Background questions</u>
Before we show you the app, we just want to spend a few minutes asking you about your cardiac device and cardiac condition.

- Do you know what kind of device you have – is it a pacemaker, is it a defibrillator, etc?
    - Why do you have this device? (I.e., what cardiac problems led to it)
    - Has anyone told you that you have something called heart failure?
    - Do you track anything, such as weight or symptoms?
        - Do you use an app or pen and paper?
        - Do you use any wearable devices?
- Do you talk to your doctor about the information they get from the device?
    - Do you have the MyLATITUDE app?
    - Have you heard of something called HeartLogic?

Intro to prototype: Now we would like to show you the prototype of the app. This prototype is just a demo so there may be things you can't click.

<table>
<tr><td>Screen</td><td>Questions</td></tr>
<tr><td colspan="2" align="center">SIGNIFICANT CHANGE</td></tr>
<tr><td>Education / onboarding</td><td>To get started, please start clicking through this flow however you would if we weren't here.
We would like you to "think aloud" as you move around the prototype. Tell us whatever is going through your mind as you go through the app. Please be totally honest because we want to know what works and what doesn't so we can make the app better for people like you.
We want to stop you there briefly to ask a few questions.
Comprehension Q: How would you explain HeartLogic in your own words? If you want to navigate back to the previous screens, let us know.
Would you watch a video to learn about HeartLogic?
What information would you hope to see in the video?
Is anything confusing? Do you have any questions?</td></tr>
<tr><td>HeartLogic dashboard</td><td>Now let's focus on this page with the HeartLogic sensors.
What is this page telling you?
Do they notice that the HeartLogic number and status?
Do they notice the color of the circle?
What would you do if you saw this page?
Try to learn if patient understands they can tap the cards to get more information.
What would you do to learn about what 22 means?</td></tr>
<tr><td>HeartLogic detail</td><td>What is this page telling you?
What does the information in the top section mean to you? (*comprehension)
What does 22 mean?
What does the color mean?
What does "significant change" mean?
Do they expand/collapse "What does this mean?"
What does the information in the graph mean to you? (*comprehension)
What is 22?
What is 6?</td></tr>
</table>

<table>
<tr><td></td><td>○ What is 16?
○ What do the background colors of the graph mean?
○ Do they click on the info icons to learn more?
■ Do they understand the information?
■ Is there anything missing?
■ What questions would they have?
● If they click on the “Why did my threshold change?” (Prompt if not)
○ What does this information mean to you?
○ Is anything confusing?
● What would you think if you saw this and weren’t feeling any symptoms?
● What would you do if you saw this information? (*behavioral intention)
○ Based on this information, would you contact your clinic/health care provider?
■ If yes, determine when – immediately, a couple of days, weeks
● Based on this information, how worried are you about worsening heart failure? (*risk)
○ Not at all, slightly, moderately, very, extremely
● What questions would you have after seeing this information?</td></tr>
<tr><td>Sensor data dashboard</td><td>● How do the sensors relate to your HeartLogic Number? (*comprehension)
● Which sensors are you interested in learning more about?
● What would you do on this page?
○ Would you click on the sensors?
● Are there any missing?
● Are any confusing?</td></tr>
<tr><td>Sensor data detail view</td><td>***PROMPT CLICKING INTO ALL SENSORS***
● What is this page telling you?
● What is being measured? (*comprehension)
○ Does participant click on “What is being measured?”
● Your [sensor name] over six months graph
○ What does this graph mean?
○ Is anything confusing?
● Is the information on this page meaningful to you?
● Is there anything missing?
● What do you do if there’s a medical word you don’t understand?
● What would you do next? (see if they can navigate back to other sensors)</td></tr>
<tr><td colspan="2" align="center">SOME CHANGE</td></tr>
<tr><td>HeartLogic dashboard</td><td>● What is this page telling you?
○ Do they notice that the HeartLogic number and status changed?
● What would you do if you saw this page?
○ Try to learn if patient understands they can tap the cards to get more information</td></tr>
<tr><td>HeartLogic detail</td><td>● What is this page telling you?
● What does the information in the top section mean to you? (*comprehension)
○ What does 12 mean?
○ What does the color mean?
○ What does “some change” mean?
○ Do they expand/collapse “What does this mean?”</td></tr>
</table>

<table>
<tr><td></td><td>

What does the information in the graph mean to you? (*comprehension)

What is 12?
What is 16?
What do the background colors of the graph mean?
Do they click on the info icons to learn more?

Do they understand the information?
Is there anything missing?
What questions would they have?

What would you do if you saw this information? (*behavioral intention)

Based on this information, would you contact your clinic/health care provider?

If yes, determine when – immediately, a couple of days, weeks

Based on this information, how worried are you about worsening heart failure? (*risk)

Not at all, slightly, moderately, very, extremely

What questions would you have after seeing this information?

</td></tr>
<tr><td colspan="2">LITTLE TO NO CHANGE</td></tr>
<tr><td>HeartLogic dashboard</td><td>

What is this page telling you?

Do they notice that the HeartLogic number and status changed?

What would you do if you saw this page?

Try to learn if patient understands they can tap the cards to get more information

</td></tr>
<tr><td>HeartLogic detail</td><td>

What is this page telling you?
What does the information in the top section mean to you? (*comprehension)

What does 4 mean?
What does the color mean?
What does “some change” mean?
Do they expand/collapse “What does this mean?”

What does the information in the graph mean to you? (*comprehension)

What is 4?
What is 16?
What do the background colors of the graph mean?
Do they click on the info icons to learn more?

Do they understand the information?
Is there anything missing?
What questions would they have?

What would you do if you saw this information? (*behavioral intention)

Based on this information, would you contact your clinic/health care provider?

If yes, determine when – immediately, a couple of days, weeks

Based on this information, how worried are you about worsening heart failure? (*risk)
Not at all, slightly, moderately, very, extremely
What questions would you have after seeing this information?

</td></tr>
<tr><td colspan="2">ADDITIONAL CONTENT</td></tr>
<tr><td>About HeartLogic</td><td>

What do you think about the information on this page?
Is this what you expected?
Is there information missing?

</td></tr>
</table>

	● Would you explore this info?<br>○ Which specifically would you want to look at?
Managing your heart health	● What do you think about the information on this page?<br>● Is this what you expected?<br>● Is there information missing?<br>● Would you explore this info?<br>○ Which specifically would you want to look at?
History	● What do you think about the information on this page?<br>● Do you want to see past data?<br>○ How far back would you want to be able to look? Why?<br>○ How often would you look at past data?<br>○ What would you do with the past data?<br>● Is there information missing?
Wrap-Up (Last 5 minutes)<br>● How do you feel after seeing the information?<br>● Do you have any questions?<br>● Would you look at this information?<br>○ *How often? (Could get repetitive to historical data questions)*<br>● What would you expect to happen now?<br>● What would you do next?	

D. Flow diagram of participant recruitment

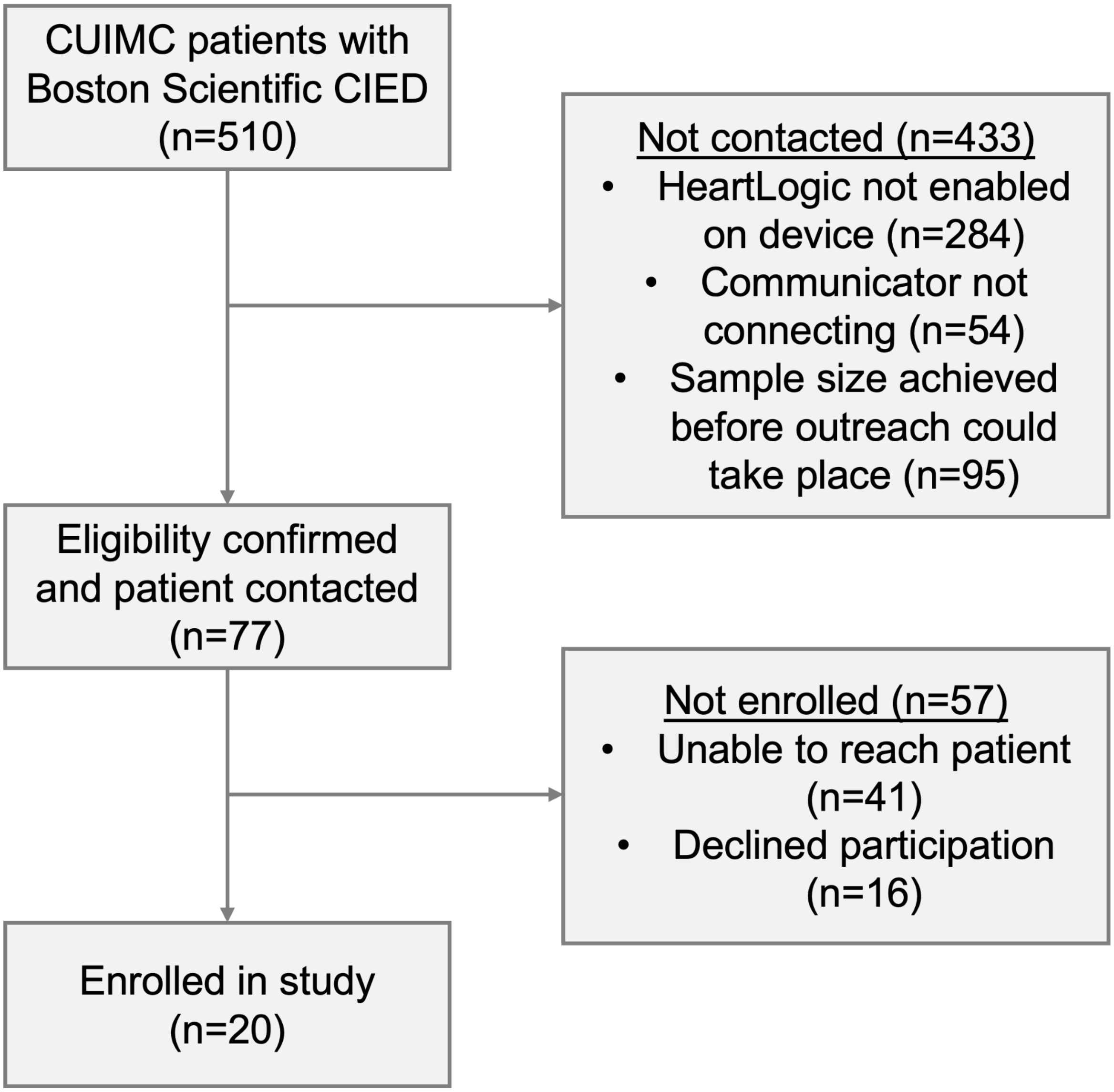

References

1. Boehmer JP, Hariharan R, Devecchi FG, et al. A Multisensor Algorithm Predicts Heart Failure Events in Patients With Implanted Devices: Results From the MultiSENSE Study. *JACC Heart Fail*. 2017;5(3):216-225.

2. Santini L, D'Onofrio A, Dello Russo A, et al. Prospective evaluation of the multisensor HeartLogic algorithm for heart failure monitoring. *Clin Cardiol*. 2020;43(7):691-697.

3. Capucci A, Santini L, Favale S, et al. Preliminary experience with the multisensor HeartLogic algorithm for heart failure monitoring: a retrospective case series report: HeartLogic algorithm for heart failure monitoring. *ESC Heart Fail*. 2019;6(2):308-318.

4. Judge S, Randall N, Goldbart J, et al. The language and communication attributes of graphic symbol communication aids - a systematic review and narrative synthesis. *Disabil Rehabil Assist Technol*. 2020;15(6):652-662.

5. Schulberg SD, Ferry AV, Jin K, et al. Cardiovascular risk communication strategies in primary prevention. A systematic review with narrative synthesis. *J Adv Nurs*. 2022;78(10):3116-3140.

6. Ancker JS, Benda NC, Zikmund-Fisher BJ. Do you want to promote recall, perceptions, or behavior? The best data visualization depends on the communication goal. *J Am Med Inform Assoc*. 2024;31(2):525-530.

7. Zikmund-Fisher BJ, Scherer AM, Witteman HO, et al. Graphics help patients distinguish between urgent and non-urgent deviations in laboratory test results. *J Am Med Inform Assoc*. 2017;24(3):520-528.

8. Desai PM, Harkins S, Rahman S, et al. Visualizing machine learning-based predictions of postpartum depression risk for lay audiences. *J Am Med Inform Assoc*. 2024;31(2):289-297.

9. Shneiderman B. Eyes Have : Task Data Type Taxonomy Information Visualizations. In: *Proceedings IEEE Symposium Visual Languages*. IEEE Computer Society Press; 1996:336-343.

10. Craft B, Cairns P. Beond Guidelines: What Can We Learn from Visual Information Seeking Mantra? Proceedings Nineth International Conference Information Visualization 2005 (IV05). Published online 2005:110-118.

11. Giddings R, Joseph A, Callender T, et al. Factors influencing clinician and patient interaction with machine learning-based risk prediction models: a systematic review. *Lancet Digit Health*. 2024;6(2):e131-e144.

12. Khullar D, Casalino LP, Qian Y, Lu Y, Krumholz HM, Aneja S. Perspectives of Patients About Artificial Intelligence in Health Care. *JAMA Netw Open*. 2022;5(5):e2210309-e2210309.

13. Benda N, Desai P, Reza Z, et al. Patient perspectives on AI for mental health care: Cross-sectional survey study. *JMIR Ment Health*. 2024;11:e58462.

14. Ancker JS, Benda NC, Sharma MM, Johnson SB, Weiner S, Zikmund-Fisher BJ. Taxonomies for synthesizing the evidence on communicating numbers in health: Goals, format, and structure. *Risk Anal*. Published online January 10, 2022. doi:10.1111/risa.13875
